# A compiler for biological networks on silicon chips

**DOI:** 10.1371/journal.pcbi.1008063

**Published:** 2020-09-23

**Authors:** J. Kyle Medley, Jonathan Teo, Sung Sik Woo, Joseph Hellerstein, Rahul Sarpeshkar, Herbert M. Sauro

**Affiliations:** 1 Department of Bioengineering, University of Washington, Seattle, Washington, United States of America; 2 Thayer School of Engineering, Dartmouth College, Hanover, New Hampshire, United States of America; 3 Department of Electrical Engineering and Computer Science, Massachusetts Institute of Technology, Cambridge, Massachusetts, United States of America; 4 eScience Institute, University of Washington, Seattle, Washington, United States of America; 5 Departments of Engineering, Microbiology & Immunology, Physics, and Molecular and Systems Biology, Dartmouth College, Hanover, New Hampshire, United States of America; Johns Hopkins University, UNITED STATES

## Abstract

The explosive growth in semiconductor integrated circuits was made possible in large part by design automation software. The design and/or analysis of synthetic and natural circuits in living cells could be made more scalable using the same approach. We present a compiler which converts standard representations of chemical reaction networks and circuits into hardware configurations that can be used to simulate the network on specialized cytomorphic hardware. The compiler also creates circuit–level models of the target configuration, which enhances the versatility of the compiler and enables the validation of its functionality without physical experimentation with the hardware. We show that this compiler can translate networks comprised of mass–action kinetics, classic enzyme kinetics (Michaelis–Menten, Briggs–Haldane, and Botts–Morales formalisms), and genetic repressor kinetics, thereby allowing a large class of models to be transformed into a hardware representation. Rule–based models are particularly well–suited to this approach, as we demonstrate by compiling a MAP kinase model. Development of specialized hardware and software for simulating biological networks has the potential to enable the simulation of larger kinetic models than are currently feasible or allow the parallel simulation of many smaller networks with better performance than current simulation software.

## Author summary

We present a “silicon compiler” that is capable of translating biochemical models encoded in the SBML standard into specialized analog cytomorphic hardware and transfer function–level simulations of such hardware. We show how the compiler and hardware address challenges in analog computing: 1) We ensure that the integration of errors due to the mismatch between analog circuit parameters does not become infinite over time but always remains finite via the use of total variables (the solution of the “divergence problem”); 2) We describe the compilation process through a series of examples using building blocks of biological networks, and show the results of compiling two SBML models from the literature: the Elowitz repressilator model and a rule–based model of a MAP kinase cascade. Source code for the compiler is available at https://doi.org/10.5281/zenodo.3948393.

## Introduction

Digital logic circuits have grown considerably in complexity since the inception of microprocessors. This growth was made possible in large part by technologies that automate the low–level record keeping, database management, routing, and placement of circuit components [1]. Digital system designers have long used hardware description languages such as VHDL (Very High Speed Integrated Circuit Hardware Description Language) and Verilog to design the logic operations of digital circuits. However, many important computing problems can benefit from analog, rather than digital, circuit design. Important examples arise from the field of biomimicry, including neuromorphic chips, which emulate biological neurons [2], and cytomorphic chips, which emulate the behavior of cellular metabolic, signaling, and genetic pathways [3, 5–11]. We have previously described programmable cytomorphic chips capable of emulating a wide range of biological reaction networks [3, 6, 8, 9, 11]. Carefully tuned cytomorphic chip–based simulations of stochastic reaction networks can achieve up to a 30,000–fold speedup over Matlab simulations [11] and a 700–fold improvement over COPASI [12, 13] on the current (prototype) hardware [6, 8, 9, 11].

However, configuring the chip for a given network currently requires manual intervention, which is a tedious process that must be repeated for every new biological pathway. Whereas many design automation tools exist for designing digital hardware, tools for the design and modeling of special–purpose analog circuits are comparatively rare. Circuit–simulation tools have been applied to neural biomimetic [14] and prosthetics [15, 16] devices and for simulating neuromorphic chips [17]. VLSI–inspired methods have been used in tools such as Cello [18] and iBioSim [19, 20], but we are unaware of any existing system which transforms a high–level biological model (a chemical reaction network) into a low–level representation for running on programmable analog hardware. We present a cytomorphic compiler—a software tool which takes as input biological pathway models encoded in the Systems Biology Markup Language (SBML) format [21] and generates a cytomorphic chip configuration as output. Our compiler provides a bridge from existing systems biology standards to cytomorphic hardware, thereby increasing the versatility of special–purpose biomimetic hardware and bringing biomimetic computing closer to practical actualization.

### Background

Models of biological networks play important roles in our understanding of disease biology [22, 23], cancer [24], drug discovery [25], metabolic regulation [26], and many other subjects. However, simulation of large kinetic network models continues to be a major challenge, despite recent progress in high–performance simulation software [27–29]. The growth in size and complexity of biological pathway models has exceeded the growth of simulation hardware and software. In one study, a whole–cell *M. genitalium* model required 10 hours on a 128 node Linux cluster in order to simulate a single cell cycle [30]. Large–scale examples of kinetic simulations also arise in genome–scale kinetic models [31, 32]. Common simulation bottlenecks arise in parameter fitting and calibration of models, which require many simulations [33]. Thus, improvements in simulation performance are necessary for better and more comprehensive model fitting and to enable larger, more robust models.

In many real–world computing tasks, the relevant metric for performance is not the total computing power of the system, but rather the computations–per–watt. A compelling example is the adoption of specialized hardware designed for Bitcoin mining, which can easily exceed 40 times the performance–per–watt of a graphics processing unit (GPU) [34]. Cytomorphic chips operate at tens of milliwatts, yet in many cases still perform equal or faster simulations than desktop computers operating at tens of watts, representing a more than 1000–fold improvement in performance–per–watt for general networks [3, 6, 8, 9, 11]. This efficiency improvement may be used to package more units onto a die, thereby allowing more simulations to run in parallel. In addition, our current prototype hardware, based on a low–yield manufacturing process, is able to achieve up to a 30,000–fold speedup over Matlab and a 700–fold improvement over COPASI for stochastic reaction networks [6].

The present work focuses on generalized, digitally–programmable cytomorphic hardware described previously [3, 6, 8, 9, 11]. The hardware maps the thermodynamic laws that govern stochastic and deterministic molecular flux in chemical reactions to stochastic and deterministic electrical current flux in electronic transistors that also obey these same laws in a mathematically exact fashion. Thus, all biological model variables, including species concentrations, parameters, and reaction rates are represented in the hardware by electric current values. However, a naïve approach at solving ODEs in this way can lead to infinite integration errors due to parameter mismatches in analog circuits, also manifest in numerical integration errors on digital computers [6, 8, 9, 11] (it is hard to easily match analog parameters to more than 10 bits of precision on digitally programmable analog cytomorphic chips whereas digital components can routinely operate at 64–bit precision, even though they are modeling biological circuits that only operate at 2–5 bits of precision [4, 8, 9, 11]).

### Key challenges

**Fig 1:**
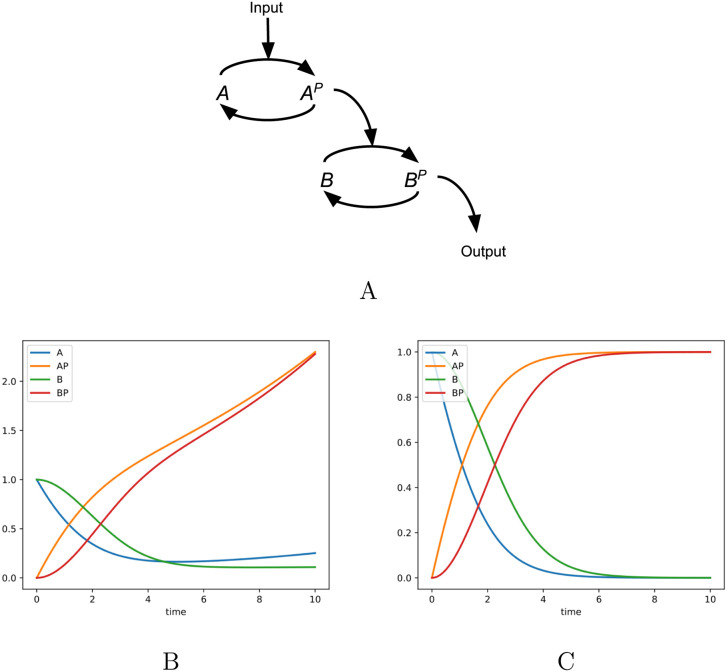
Demonstration of the divergence problem. In the phosphorylation cascade in (A), the quantities *A* + *A*^*P*^ and *B* + *B*^*P*^ should be constant in time. However, letting $k_{fA}^{\prime }=k_{fA}+\alpha$ and $k_{rA}^{\prime }=k_{rA}-\alpha$ results in the loss of this conservation relationship, as shown by the value of *A*^*P*^ in the numerical integration of this ODE system (B), which exceeds the total starting amount of *A* + *A*^*P*^ = 1. This phenomenon also applies to networks that do not have conserved quantities, as any steady–state value will tend to drift over time. Using the total quantity representation of Eq (2), this problem can be eliminated (C).

A major challenge in simulating biochemical models is ensuring flux symmetry (i.e., when species *A* is converted to species *B*, the rate of production of *B* should be *exactly* the rate of consumption of *A*). Consider the situation shown in Fig 1. This figure depicts a kinase cascade the active form of kinase *A*, represented as *A*^*P*^, which in–turn phosphorylates kinase *B*, with their corresponding rates of change:

$$\begin{array}{c} \begin{array}{cc} \frac{dA}{dt} & =-k_{fA}\frac{A}{1+A}+k_{rA}A^{P}\\ \frac{dA^{P}}{dt} & =k_{fA}^{\prime }\frac{A}{1+A}-k_{rA}^{\prime }A^{P}\\ \frac{dB}{dt} & =-k_{fB}A^{P}\frac{B}{1+B}+k_{rB}B^{P}\\ \frac{dB^{P}}{dt} & =k_{fB}^{\prime }\frac{B}{1+B}-k_{rB}^{\prime }B^{P} \end{array} \end{array}$$ (1)

Since the total amount of the kinase *A* is constant, the rate of change of *A*+ *A*^*P*^ should be zero. However, consider the case where perturbations are added to the kinetic constants for production of of *A*^*P*^ (letting $k_{fA}^{\prime }=k_{fA}+\alpha$ and $k_{rA}^{\prime }=k_{rA}-\alpha$) and *B*^*P*^ ($k_{fA}^{\prime }=k_{fA}+\beta$ and $k_{rA}^{\prime }=k_{rA}-\beta$). It can be seen from these equations that this will only occur if either *α* and *β* are zero, or $\frac{A}{A^{P}}=\frac{\beta }{\alpha }$. However, the steady state values of *A* and *A*^*P*^ are already fixed by the equilibrium ratio of the first step in the cascade. Therefore, for general perturbations *α* and *β* the quantity *A* + *A*^*P*^ will change over time, violating conservation laws. In this example, we considered a kinase cascade because it shows how the divergence problem clearly violates conservation laws, but this phenomenon actually applies to all networks that reach a steady state, regardless of whether conserved quantities exist in the network or not.

To address this problem, the cytomorphic chip is designed to operate on conserved quantities of the system, which are mapped to conservation laws such as Kirchhoff’s current law in electronic circuits, a law that is always precisely obeyed [6, 8, 9, 11]. In the example in Fig 1, the conserved quantities are *A* + *A*^*P*^ = *A*_*tot*_ and *B* + *B*^*P*^ = *B*_*tot*_. The system can be described using a pair of differential equations corresponding to *A*^*P*^ and *B*^*P*^:

$$\begin{array}{c} \begin{array}{cc} \frac{dA^{P}}{dt} & =k_{fA}^{\prime }\frac{A_{tot}-A^{P}}{1+(A_{tot}-A^{P})}-k_{rA}^{\prime }A^{P}\\ \frac{dB^{P}}{dt} & =k_{fB}^{\prime }\frac{B_{tot}-B^{P}}{1+(B_{tot}-B^{P})}-k_{rB}^{\prime }B^{P}\\ A & =A_{tot}-A^{P}\\ A & =B_{tot}-B^{P} \end{array} \end{array}$$ (2)

### Chip layout

Here, we briefly review the layout and specifications of the cytomorphic chip. A more complete description of the hardware can be found in [6, 8, 9, 11].

**Fig 2:**
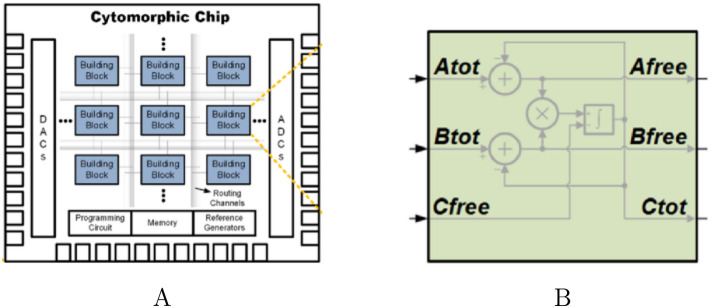
Layout of the cytomorphic chip. The chip is composed of 20 blocks of reaction units (A). Each block accepts as input total quantities for reactants *A*_*tot*_ and *B*_*tot*_ and outputs the total amount of product *C*_*tot*_, as well as any remaining “free” reactant *A*_*free*_ and *B*_*free*_ which has not yet been converted into product. These current–based inputs and outputs are converted into digital signals before being sent outside the chip in order to prevent signal degradation.

Fig 2 depicts the layout of the cytomorphic chip. The chip is composed of 20 blocks, each designed to solve a single biochemical reaction of the form:

$$\begin{array}{c} A+B⇌C+D \end{array}$$

where the rate of this reaction is *k*_*f*_ * *A* * *B* − *kr* * *C* * *D*. In practice, modelers are accustomed to working with more complex reactions with lumped kinetic expressions such as Michaelis–Menten kinetics. However, physical processes at the molecular level invariably fall into this binary mass–action category. In protein complex formation, subunits are added one–at–a–time, and in enzyme catalysis, substrates bind the enzyme in an intermediate state. We will show later how lumped kinetic expressions can be used with the hardware.

## Methods

**Fig 3:**
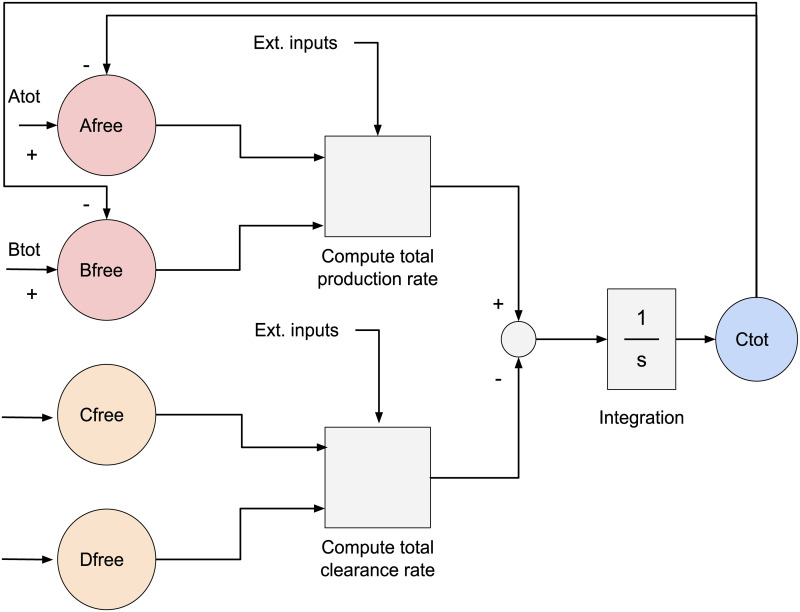
A flow diagram for the cytomorphic compiler. The compiler processes the input SBML model to “expand” (see below) lumped kinetic expressions into constituent bimolecular elementary processes. Elementary reactions are then mapped to blocks on the chip (sometimes to multiple blocks, as in fan–out reactions described below). Each block is assigned parameter values based on the forward and reverse rate constants of its respective reaction, and potentially degradation of the product. Blocks are connected together based on the topology of the reaction network, but care must be taken to maintain a single “total” value for each species, as described in the “Network Building Blocks” section. The final output of the compiler is a configuration for the shift registers (which store parameter values) for all used blocks and SRAM (which connects block input and output ports).

Fig 3 shows a high–level overview of the compiler. The compiler accepts as input a SBML [35] model parsed using JSBML [35, 36], or an Antimony file [37], which is a human–readable format directly interconvertible with SBML. SBML containing arbitrary rate laws cannot be run on the cytomorphic hardware. We have devised a method of “expanding” lumped kinetic expressions such as Michaelis–Menten, Botts–Morales, and repressor binding kinetics that allows networks using these formulations to be compiled onto the hardware.

The output of the cytomorphic compiler is two files: configuration of the shift registers (which specify the parameters of each block), and the SRAM (which specifies connections between blocks). We cover each of these file types below.

### Terminology & validation methods

Table 1 lists definitions for terminology used in this article.

**Table 1:** Terminology used in this article.

Term	Description
**Cytomorphic Chip**	A single chip with 20 reaction blocks.
**Antimony**	A human–readable and writable representation of SBML.
**Block simulation**	A circuit–level simulation (performed on a computer) of a specific configuration of the cytomorphic chip (including parameters and connections). This simulation is based on the ODE model of Fig 4, which shows the transfer function for every component in the block. A block simulation can be performed using either Simulink or libroadrunner [27]. In either case, the files to run the block simulation are generated by the compiler.
**Kinetic expansion**	Classic enzyme kinetics like the Michaelis–Menten rate law are based on lumped processes. A Michaelis–Menten process represents substrate binding and catalysis in one step but these are mechanistically two separate processes. The cytomorphic compiler breaks these lumped expressions down into their constituent components, using lumped constants such as the Michaelis constant *K*_*M*_ to determine rate constants.
**Archetype**	We use this term to denote the canonical form of a rate law expression such as the Michaelis–Menten formula $\frac{V_{max}S}{K_{M}+S}$. See the section “Matching Algorithm for Lumped Kinetics” below.

**Fig 4:**
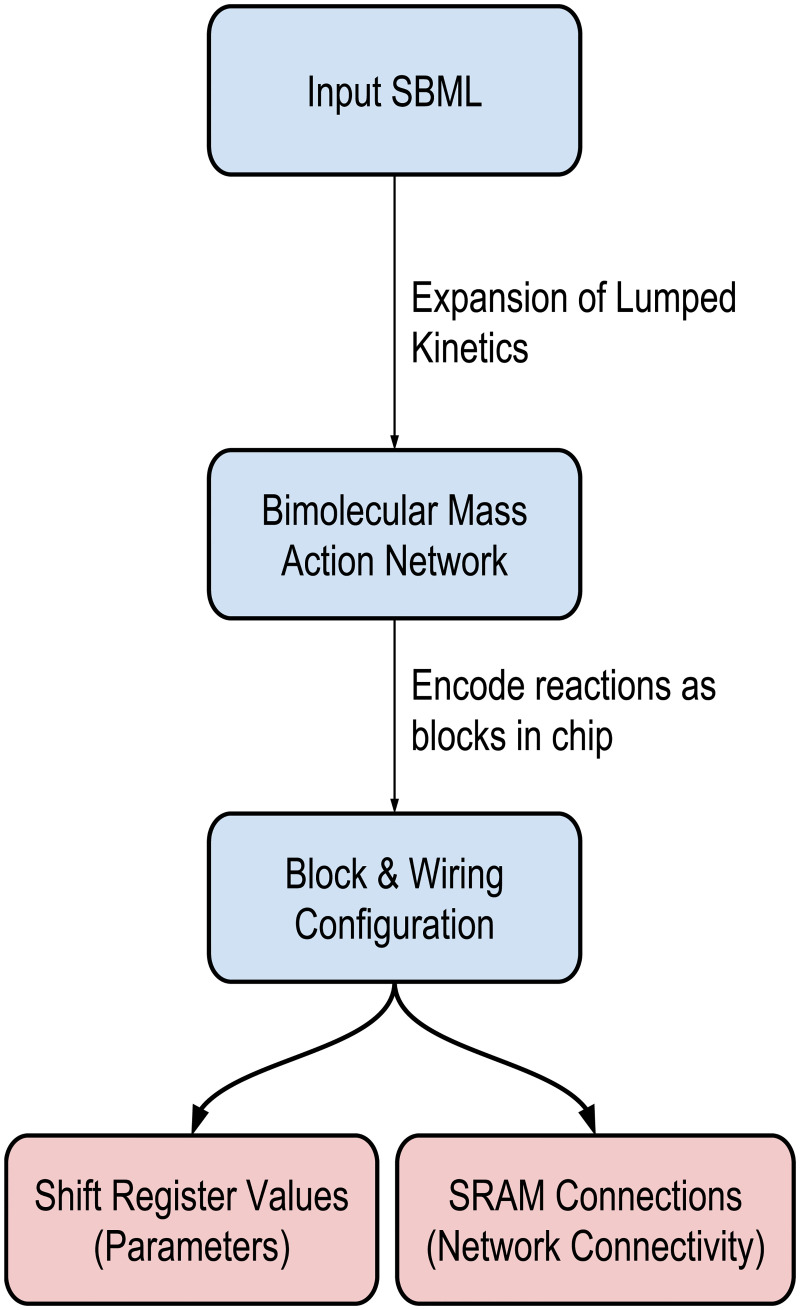
Simplified conceptual block diagram of a reaction unit (see [9, 11] for a detailed circuit–level diagram). The chip has a total of 20 reaction units. Each reaction units block accepts as input total quantities for reactants *A*_*tot*_ and *B*_*tot*_ and outputs the total amount of product *C*_*tot*_, as well as any remaining “free” reactant *A*_*free*_ and *B*_*free*_ which has not yet been converted into product. These current–based inputs and outputs are converted into digital signals before being sent outside the chip in order to prevent signal degradation.

In addition to producing programming files for the cytomorphic hardware, the cytomorphic compiler produces two other types of output that can be used to validate the compiler: (1) A Simulink model containing the blocks, parameters, and connections produced by the compiler, or (2) a differential equation system called a *block simulation* based on the block diagram of Fig 4. Either of these outputs can be used to simulate the circuit behavior over time, similar to the SPICE analog circuit simulator [38] used in circuit design, except that the simulations are transfer function–based (i.e. they are based on the block diagram of Fig 4, which uses gains, multipliers, and summation blocks for each stage instead of individual circuit components). In fact, these two formats are numerically equivalent, but serve different use cases. We use Simulink diagrams to visualize the block wiring, whereas we use block simulations to plot and compare compiler output in Jupyter notebooks. In the case of mass–action networks, the block simulation should correspond exactly to the SBML simulation. However, the underlying differential equations in the block simulation are based on total quantities, whereas SBML uses free quantities.

When evaluating the performance of the cytmorphic chip against conventional software simulation, it should be noted that some practical limitations exist in the current prototype of the hardware, which makes exact quantitative comparison with software simulations challenging. These include manufacturing variations in resistance / capacitance, the capacitance of the block integrator, analog–to–digital converter (ADC) clock speed and transistor mismatch. For variations that can be measured, we calibrate the compiler to adjust the parameters based on the magnitude of the variation. As described in detail later and as shown in S7 Fig, we can compensate for most of the output variation between the expected output and the output generated by the chip by adjusting the internal gains.

Despite the lower precision of analog simulations as compared to software simulation, it is often sufficient for running biological simulations. For example, Proctor et al. present simulations of the two stochastic models of the p53 signalling pathway [39]. The authors focus on whether their proposed models predict 1) the “existence” of sustained oscillations, 2) the shape of oscillations, 3) the effect of parameter changes or perturbations, and 4) the effect of stochasticity. In doing so, less attention is paid to the precise values of oscillation period, molecular concentrations, or parameters, partly because many of them are highly variable or not known. It is also because biological networks are often quite robust to variations in parameters.

### Shift registers & block parameters

For the default configuration of the FF_EN_sw switches in Fig 4, it is apparent that the rate of production of the block’s main product, *C*, is given by:

$$\begin{array}{c} \begin{array}{cc} \frac{dC}{dt} & =k_{r}((A_{tot}-C_{tot}\cdot A_{FB\_EN}){\left(\frac{B_{tot}-C_{tot}\cdot B_{FB\_EN}}{KD_{fw}}\right)}^{n}+C_{prod}\\ & -(\frac{C_{free}D_{free}}{KD_{rv}}))-k_{deg}(C_{free}\left(ratC\right)+C_{deg}) \end{array} \end{array}$$ (3)

The forward and reverse rates for each block are determined by a combination of *k*_*r*_ and either *KD*_*fw*_ or *KD*_*rv*_. Examining the equation shows that the forward and reverse rate constants are given by $\frac{k_{r}}{KD_{fw}}$ and $\frac{k_{r}}{KD_{rv}}$ respectively. Thus, the forward and reverse dissociation constants can be used to tune the relative forward and reverse rates, whereas the overall reaction rate of all blocks in the network can be tuned by changing the *k*_*r*_ value. Also, any block that produces a species can also serve as a degradation reaction by utilizing *ratC*.

### SRAM & network building blocks

The chip’s SRAM is used to program connections between the input and output ports of the blocks. While each protein block is designed to simulate a fundamental second-order biochemical reaction, we can capture more complex dynamics by connecting several blocks together. Since it is instructive to visualize how total variables affect block connection in non-trivial ways, we demonstrate topologies involving multiple blocks that simulate a 3-stage feed-forward network cascade and a “fan-out” reaction. These reactions are chosen because they form the basis for most larger networks and exhibit several recurrent wiring topology that appear in more complex building blocks. These building blocks are selected from elementary types of subnetworks—they are not related to motifs (which are defined by statistical over–representation in real networks [40, 41]). Instead, building blocks are comprised of simple subnetworks such as “fan–in” and “fan–out” configurations, where a single species is produced or consumed by multiple reactions respectively. For a more in-depth discussion on these circuit networks, we refer interested readers to previous publications [8, 9].

**Fig 5:**
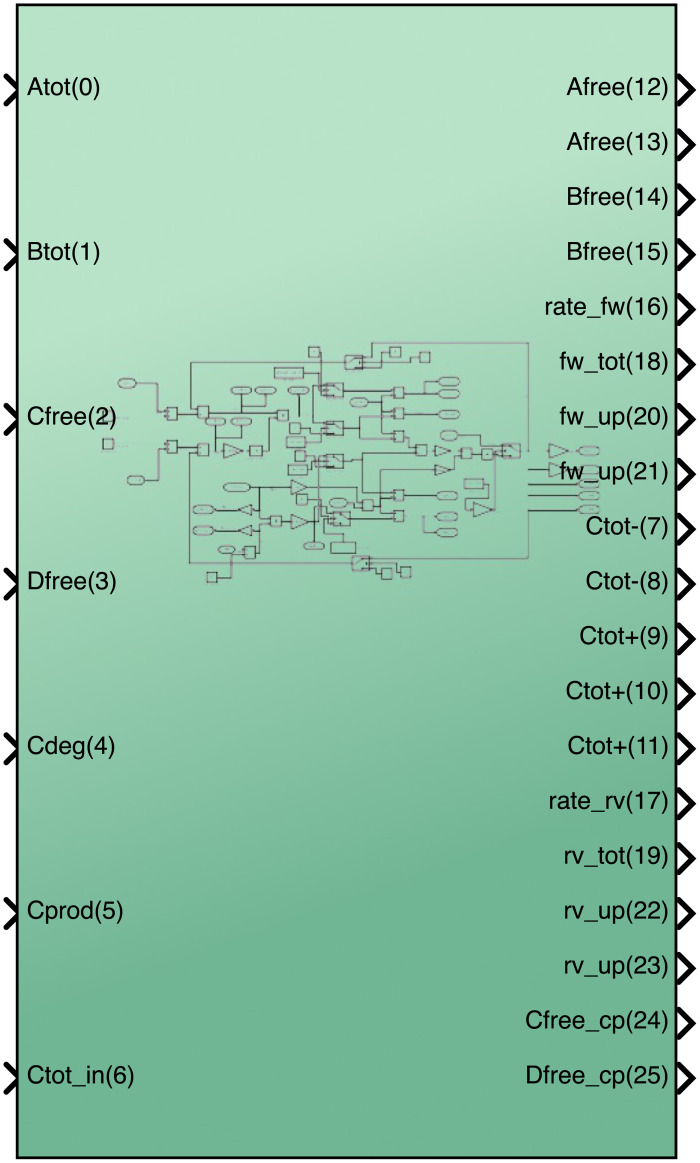
Input and output ports for the cytomorphic block. The two main inputs *A*_*tot*_ and *B*_*tot*_ are used to compute the forward rate. Internally, the block subtracts its own *C*_*tot*_ value from *A*_*tot*_ and *B*_*tot*_ to compute *A*_*free*_ and *B*_*free*_ (see Fig 2B). The chip’s main output is *C*_*tot*_. If there is another reaction that consumes *C*, then *C*_*tot*_ should be connected to the *A*_*tot*_ or *B*_*tot*_ input of the consumer block. Otherwise, *C*_*tot*_ should be connected to the block’s own *C*_*free*_ input to allow calculating the reverse rate. If the reaction is reversible, *D*_*free*_ may also receive input from another block or be simply wired to unity in the case of a single product *C*. Other ports describing the blocks forward and reverse rates are used in in certain building blocks described below. The chip has 2 copies of Afree, Bfree, fw_up, rv_up, and 5 copies of Ctot (2 negative and 3 positive). In addition, the chip also copies the values of its *C*_*free*_ and *D*_*free*_ inputs to the Cfree_cp and Dfree_cp output ports respectively. These copies are used to route the input values to additional blocks in fan–in configurations (see below).

Fig 5 is a graphical abstraction of a single protein block. The currents flowing into the input ports represent the concentration of reactants in a forward reaction. The currents flowing out of the output ports represent the concentration of products formed from the reaction. Since each reactant and product may participate in several downstream reactions, the concentration currents for Afree, Bfree and Ctot are copied using current mirrors. In what follows, we use species names S, T, etc. to distinguish species in the network from the port names on the block (A, B, C, and D).

**Fig 6:**
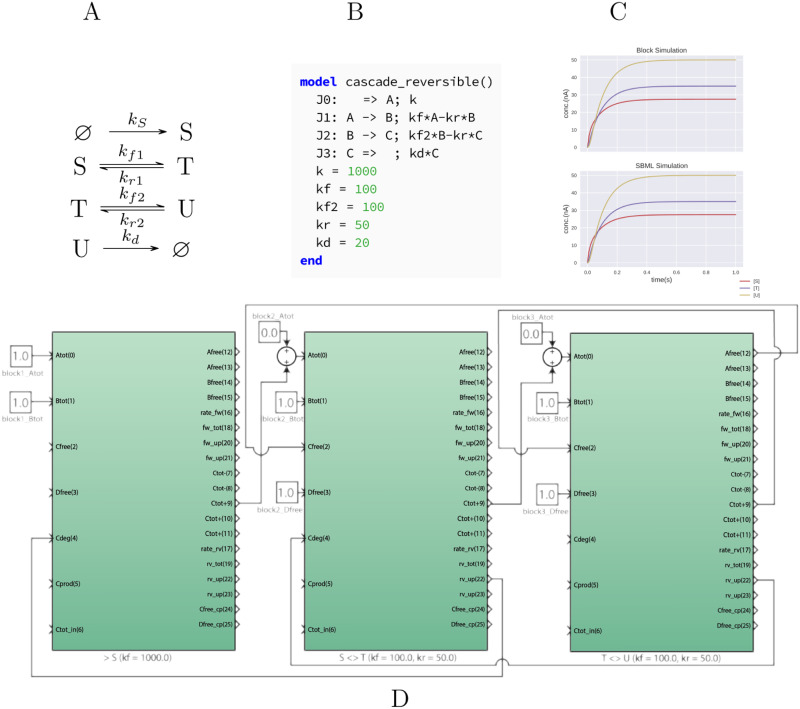
A three–step feed–forward network. This network is comprised of a linear chain of unimolecular mass–action processes (A). To convert this network to a wiring, we designate blocks 1–3 as the main produces of species *S*, *T*, and *U* respectively. Block 1 produces *S*, hence its main output port *C*_*tot*_ is connected to the *A*_*tot*_ input of block 2 and so on. This signal is summed with the initial value of *T* (if non–zero). Since the cytomorphic chip uses currents for computation, summing signals is achieved simply by connecting multiple signals to the same input port. The last block in the chain, which produces *U*, also serves as a degradation reaction $U\overset{k_{d}}{\to }⌀$. The block has its ratC parameter set to the degradation rate *k*_*d*_. Additionally, the amount of *U* degraded must also be subtracted from the total values of *S* and *T*. The rv_up port computes the total loss in *C* for each block and is propagated to the block immediately upstream by connecting to the Cdeg port. The Antimony/SBML model for this network (B) is converted to a block simulation that gives identical output to the SBML simulation (C). Since this is a mass–action network, numerical differences can be made arbitrarily small by adjusting integrator tolerances. All connections created by the compiler are shown in the wiring diagram. More info is available at https://github.com/cytocomp1/cytocomp/tree/master/case-studies.

To simulate a 3-stage feed–forward biochemical cascade, we connect three protein blocks to simulate a single irreversible production reaction, two reversible substitution reactions, and an irreversible degradation steps as shown in Fig 6. We need just three protein blocks instead of four to simulate these reactions because each block is designed to simulate both a second order reaction and product degradation. In this case, block 3 is wired to simulate both the substitution reaction between T and U and the degradation of U. To represent the production of S and its conversion to T, we connect Ctot of block 1 to Atot of block 2. This connection is repeated between block 2 and 3 for the substitution reaction of T to U. To simulate the reverse reaction fluxes, we connect output ports that represent the concentration of free products to input ports Cfree to produce the right concentration of free species. Finally, to account for the decrease in total variable of species S and T as U degrades, we backpropagate the degradation flux of U upstream by connect ports rv_up from block 3 and 2 to ports Cdeg from block 2 and 1 respectively. These design principles hold true for larger feed-forward circuit cascade as well.

In the “fan–out” reaction shown in S1 Fig, two protein blocks were used to model the two reversible reactions. Similar to the feed-forward cascade, ports Cfree on block 1 and 2 are wired to ports Ctot so that the free concentration of U and W can be used to simulate the correct reverse flux. Since there is no degradation reaction, we do not need to backpropagate any loss of product via rv_up. Instead, to calculate the unbound concentration of S, we simply subtract U and W from input which is achieved by closing an internal use-it-and-lose-it feedback loop in block 1 and adding a negative current Ctot of block 2 respectively. We have included two additional examples for a dissociation reaction and a “fan–in” reaction in the supporting information (S2 and S3 Figs).

### Lumped kinetics

The term “lumped kinetics,” as used here, refers to any kind of process in a model that represents multiple elementary steps, where an elementary step is defined as a bimolecular mass action reaction (e.g. substrate binding / unbinding, or the catalytic step in enzyme catalysis). A common instance of this is enzyme kinetics: *E* + *S* ⇌ *ES* → *E* + *P*, which represents an enzyme *E* that converts substrate *S* into product *P*. These reactions occur at the following rates:

$$\begin{array}{c} \begin{array}{cc} E+S⇌ES, & \;k_{f}E\cdot S-k_{r}ES\\ ES\to E+P, & \;k_{cat}ES \end{array} \end{array}$$ (4)

The individual forward and reverse rate constants of the binding step are difficult to measure directly, so these two processes are usually condensed into a single process by assuming either rapid equilibrium of the binding process (which leads to the well–known Michaelis–Menten kinetics [42]) or by assuming the enzyme–substrate complex is at steady–state (Briggs–Haldane kinetics [43]). In either case, the rate law for the resulting lumped process can be expressed as:

$$\begin{array}{c} k_{cat}E\frac{S}{K_{M}+S} \end{array}$$

where $K_{M}=\frac{k_{r}+k_{cat}}{k_{f}}$ for Briggs–Haldane kinetics or *k*_*r*_/*k*_*f*_ for Michaelis–Menten kinetics.

Our general approach to simulating these lumped expressions on the cytomorphic chip is to break them down into the constituent steps of Eq 4. However, this re-creates the problem of determining the forward and reverse rate constants *k*_*f*_ and *k*_*r*_ respectively. From the lumped expression, we can determine *k*_*cat*_ and *K*_*M*_. However, we need one additional constraint to specify both *k*_*f*_ and *k*_*r*_. This constraint comes from the upper limit of the chip’s simulation speed.

**Fig 7:**
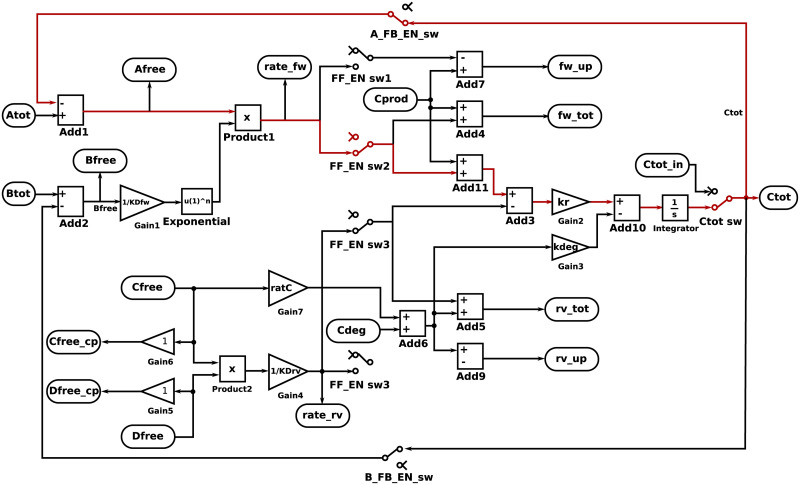
Negative feedback around the circuit for *A*.

Consider the block diagram of a reaction unit containing a negative feedback loop around the part of the circuit that processes *A* as highlighted in Fig 7. In designing electronic amplifiers, it is common to account for the so–called *phase margin*. In an amplifier circuit as well as in the highlighted feedback loop, there exists the possibility that the output signal can be 180° out–of–phase with the input. Since the feedback is negative, the signal will be inverted and cause constructive interference with the input, leading to instability. Furthermore, this feedback loop also possesses a parasitic pole due to the current mirror that produces the *A*_*free*_ signal. Taken together, these conditions lead to the stability rule [11]:

$$\begin{array}{c} \frac{B_{tot}}{KD_{fw}}\frac{r}{C}<\frac{A_{free}}{C_{par}} \end{array}$$

where *C*_*par*_ is the parasitic capacitance at the gate node of the *A*_*free*_ current mirror, *C* = 0.1*μF* is the capacitance of the integrator capacitor, and *r* is the overall rate of the block (used as a scaling factor for both the forward and reverse rates). In the preceding expression, we assumed that *A*_*free*_ < *B*_*tot*_. The roles of *A* and *B* can be reversed if this is not the case. This equation can be simplified to:

$$\begin{array}{c} k_{f}<\frac{A_{free}}{B_{tot}}\frac{C}{C_{par}}=\rho \end{array}$$ (5)

This gives an upper bound for the value of *k*_*f*_ based on the global value $\frac{C}{C_{par}}$ and the local value $\frac{A_{free}}{B_{tot}}$ which depends on the reaction and simulation conditions. These two values can be condensed into a single constant *ρ*, called as the margin, which in general varies per reaction. We will encounter several more examples of lumped expressions that expand into elementary processes that occur at rate *ρ*. In practice, we can assume a reasonable lower bound for *ρ* and refine the estimate after simulating the network. If we assume *C*_*par*_ = 1*pF*, this gives an upper–bound of $10^{5}\frac{A_{free}}{B_{tot}}$. The value of $\frac{A_{free}}{B_{tot}}$ is model and simulation–dependent, but by assuming a reasonable upper bound of 100, we obtain *ρ* = 1000. This value can be used to run digital simulations for a given hardware configuration which in turn can be used to refine the value *ρ*.

Returning to enzyme kinetics, the higher the value we choose for *k*_*f*_ (and hence *k*_*r*_), the more rapid the enzyme–substrate binding. Since Michaelis–Menten kinetics are derived based on an equilibrium assumption, a larger *k*_*f*_ will tend to make this assumption more valid. Therefore, choosing $k_{f}\approx \min(\frac{A_{free}}{B_{tot}})\frac{C}{C_{par}}=\rho$ yields the best approximation to Michaelis–Menten kinetics without causing instability.

**Fig 8:**
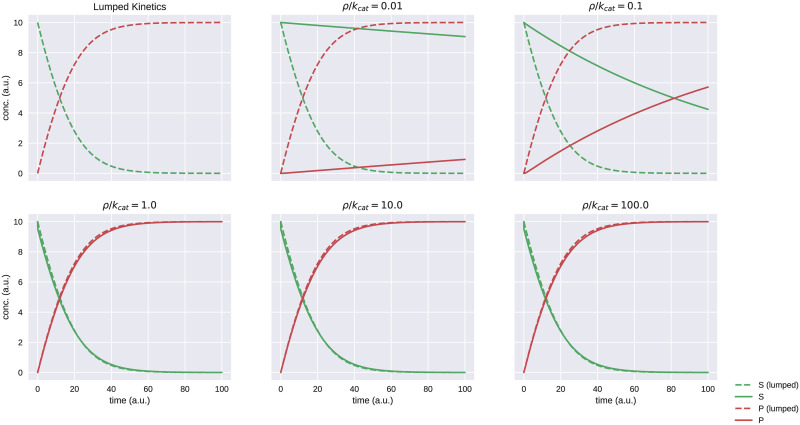
Different margin values and their respective simulations. In these simulations, *E* = 1, *S*_*initial*_ = 10, *k*_*cat*_ = 1, and *K*_*M*_ = 10. For the elementary two–process network, the reverse binding rate is calculated automatically by the compiler using the supplied value of *ρ* as *k*_*r*_ = *ρK*_*M*_ − *k*_*cat*_ if $\frac{k_{cat}}{k_{r}}⪡1$ or *k*_*r*_ = *ρK*_*M*_ otherwise.

Fig 8 shows the result of plotting the dynamics of the elementary binding / catalysis network versus the original Michaelis–Menten lumped single–process network for various values of *ρ*.

It is worth observing that, from a design standpoint, our compiler uses the two–step network to “approximate” models with lumped processes such as Michaelis–Menten kinetics. However, from a mechanistic standpoint, Michaelis–Menten kinetics represents an approximation of the corresponding physical two–step process. In effect, this line of reasoning has taken us full-circle from a mechanistic representation to a lumped process and back again. However, for the purpose of practical modeling, this is a necessary detour, since it is feasible to measure the *K*_*M*_ and *V*_*max*_ values for enzymes, but in general it is not possible to measure binding apart from catalysis (i.e. the parameters *k*_*f*_, *k*_*r*_, and *k*_*cat*_).

The meaning of this observation is that while elementary, mass–action processes are biologically accurate, they are not feasible from a modeling standpoint. Thus, modelers should continue to use kinetics that can be parameterized with quantifiable parameters, while specialized hardware should continue to use elementary processes that are conducive to efficient implementation. Breaking–down these high–level expressions into low–level expressions, what we hereafter refer to as “expansion”, is one of the main functions of the cytomorphic compiler.

A limitation of this “expansion” method is that when the substrate is not in excess of the enzyme (i.e. *E* ≪ *S* does not hold), then there is significant deviation between the two–step mechanistic process and the idealized Michaelis–Menten approximation (the mechanistic process will tend to lag behind the lumped process), regardless of the value of *ρ*. However, Michaelis–Menten kinetics also relies on the assumption that *E* ≪ *S*, and thus would be a physically inaccurate modeling assumption in this case.

### Matching algorithm for lumped kinetics

In order to successfully expand lumped kinetic expressions into constituent components, it is necessary to (1) identify, from the rate law, what type of lumped expression is represented, and (2), obtain the values of all lumped constants and use these to compute the individual rate constants.

One approach to solving (1) would be to simpify and canonicalize the rate law expression and compare this simplified version with each known kinetic expression on a tree–basis (we refer to canonical expressions like the Michaelis–Menten formula $\frac{V_{max}S}{K_{M}+S}$ as “archetypes”). However, this approach is sensitive to different factorizations of the expression and requires that all archetypes also be in canonical form. Instead, we have opted to use an algorithm for determining the equivalence of expressions based on hash–coding [44].

**Fig 9:**
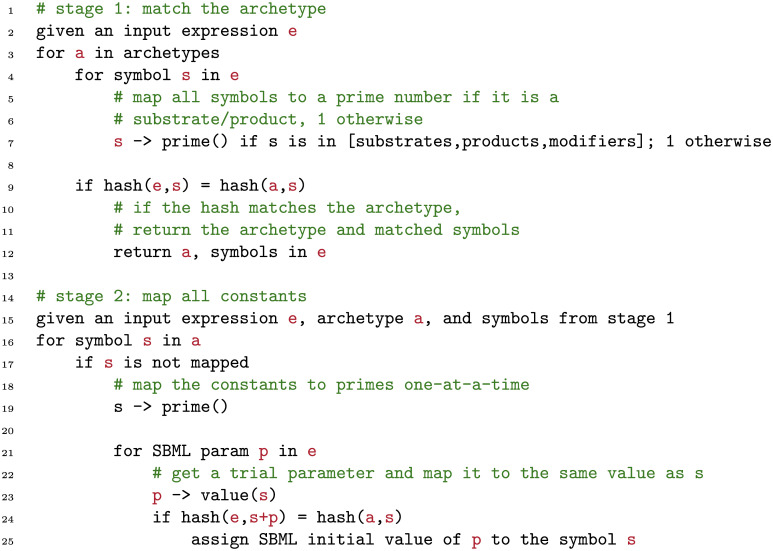
Pseudocode for the Martin matching algorithm.

In 1971, William Martin observed that, by assigning numeric values to symbols appearing in a mathematical expression, an equivalence class can be computed for a given expression by simply evaluating it on a finite field. Two mathematical expressions can thus be evaluated for equivalence by simply comparing their “hash codes,” so–constructed. Based on this observation, we have constructed an algorithm that operates on a database of common rate–laws and their “hash codes.” We call these common rate–law expressions *archetypes*, and pre–compute their hash codes according to the algorithm below. We then compute these pre–computed values to input expressions to determine which type of lumped kinetics a reaction employs. The algorithm is split into two stages (lines 1–12 and 14–25 in Fig 9 respectively).

The first stage accepts as input an SBML kinetic law expression in the form of an abstract syntax tree (AST). The leaves of this tree may be either the substrate / product species that participate in the reaction, or symbols that refer to numerical constants (such as *K*_*M*_). The algorithm assigns a different prime number to each symbol in the AST if it is a substrate / product of the reaction (Fig 9 line 7) and unity otherwise. Symbols that are assigned relatively prime values may be used in finite field expressions (such as addition, multiplication, etc.) and the results will still contain relatively prime factors, which helps to distinguish the hash code values after many operations.

On the other hand, when constructing archetypes, any symbolic constant (such as *K*_*M*_) can in principal contain any value and any symbolic name in a given SBML model. Hence, we assign a value of 1 to these constants. Hence, we must also assign a value of 1 to all constants in the matching phase.

The first stage then computes the hash code of the expression to the known values for all archetypes in the database according to Martin’s algorithm (Fig 9 line 9). The output of the first stage is the matched archetype along with the positions of the substrates and products in the expression (Fig 9 line 12).

Once the input expression has been mapped to a valid archetype, we can proceed to map kinetic constants within the expression (for example, to sort out which symbols in the input expression correspond to *K*_*M*_ versus *V*_*max*_). For each constant, we assign a numeric value, setting all other constants to unity (Fig 9). The assigned constant, as well as all substrates and products, must be relatively prime. We then iterate through all SBML parameters (Fig 9 line 21) and substitute the same prime number for the value of this parameter in the input expression 9 line 23). If the computed hash of the input expression matches the archetype, the parameter *p* in the input expression corresponds to the symbol *s* in the archetype and we identify the role of *p* as being either *K*_*M*_, *V*_*max*_, etc. in the returned datastructure.

It is evident from Fig 9 that the complexity of the first stage of the matching algorithm is *O*(*N*_*s*_*N*_*a*_), where *N*_*s*_ is the number of symbols in the input expression, and *N*_*a*_ is the number of archetypes in the database. Fortunately, most kinetic models make use of a limited repertoire of kinetic laws, such as Michaelis–Menten, Botts–Morales, and Monod–Wyman–Changeaux kinetics. This limits both *N*_*s*_ and *N*_*a*_ in most practical models.

The second stage of the algorithm has *O*(*N*_*p*_*N*_*a*_) complexity, where *N*_*p*_ is the number of symbols in the input expression which map to SBML parameters, and *N*_*a*_ is the number of symbols in the archetype *a*. For the same reasons as above, these are also capped in most practical models.

## Results and discussion

### Gene regulatory kinetics

Another major type of lumped kinetics occurs in models of gene regulatory networks. Consider the LacI repressor, which controls expression of the *lac* operon (*lacO*) in bacteria. The LacI repressor is a homotetramer, but might be better described as a dimer of dimers. Each dimer subunit contains a DNA–binding site for *lacO*. The repressor binds to *lacO* as a two–step process. Binding of allolactose to LacI causes the repressor to enter an inactive state P (protruded) with decreased overall affinity, releasing the operator site. Using lumped kinetics, the transcription rate of the operator is given by [45, 46]:

$$\begin{array}{c} \nu =\alpha_{0}+\alpha \frac{K_{m}^{2}}{K_{m}^{2}+R^{2}} \end{array}$$

where *ν* is the transcription rate, *α* is an experimentally determined rate constant, *K*_*m*_ is the equivalent equilibrium constant of the two binding steps, *R* is the concentration of the active repressor, and *α*_0_ is the basal level of transcription under fully repressed conditions (the “leakage” rate).

In order to expand this process to a simulatable form, we could decompose binding into a two–step process. However, the cytomorphic chip provides a Hill function that allows this two–step process to be modeled as a single step (labeled *n* in Fig 2). Using the Hill function, we could write the overall binding process as:

$$\begin{array}{c} R+O⇌B,\;k_{f}^{2}R^{2}O-k_{r}B \end{array}$$

where *R* is the active repressor, *O* is the operator site, and *B* is the bound (repressed) complex. Transcription can then be modeled as a simple first–order process without substrate depletion (both the A_FB_EN and B_FB_EN in Table 2 should be off). This expanded model exhibits a time delay proportional to *τ* = *kr* + (*k*_*f*_ ⋅ *R*)^2^ compared to the lumped expression, which assumes rapid equilibrium of the binding process. Our approach to minimizing this discrepancy is the same as in the enzyme kinetics case—we maximize the forward rate constant *k*_*f*_ subject to the margin *ρ* defined in Eq 5. The expanded model can reproduce the dynamics of complex networks to a high degree of accuracy, as shown below.

**Table 2:** Tunable parameters for each block.

Parameter	Description
ratC	Controls the degradation rate of the main product *C* when this block also serves as a degradation reaction.
n	Hill coefficient for forward binding. Useful in repressor kinetics.
KDfw	Forward–binding dissociation constant. Used to specify the forward rate via Eq 3.
KDrv	Reverse–binding dissociation constant. Used to specify the forward rate via Eq 3.
kr	The overall rate of the block. Can be used to tune the forward and reverse rates simultaneously (trading speed for stability or vice–versa).
kdeg	Auxiliary degradation rate used in the fan–in configuration.
A_FB_EN,B_FB_EN	Substrate depletion switches for reactants *A* and *B* respectively. When turned off, product of *C* does not consume *A* or *B* resp. (useful for modeling transcription and translation reactions).
FF_EN_sw1,2,3,4	Switches controlling the internal forward–reverse rate computation of the block. Only used in fan–in configurations, which each block must subtract the “main” production rate from its own rate.
Ctot_sw	A switch controlling whether the block’s main output Ctot is supplied externally (not used in most configurations).

Programmable parameters for each cytomorphic block.

In a highly influential study in 2000, Elowitz *et al* showed that a genetic oscillator (the “repressilator”) can be constructed from three genetic repressors [45]. To validate the cytomorphic compiler’s ability to translate repressor kinetics, we obtained a dynamical model of the repressilator from the BioModels database [47, 48]. This model contains a total of 12 reactions, half of which are degradation reactions. To reduce the number of blocks required to encode the model, we condensed the degradation reactions into the production rate laws for the three genes and proteins in the system:

$$\begin{array}{cc} ⌀\to X,\; & \alpha_{0}+\alpha \frac{K_{m}^{2}}{K_{m}^{2}+R^{2}}-k_{d1}X \end{array}$$ (6)

$$\begin{array}{cc} ⌀\to P_{X},\; & k_{t}X-k_{d2}P_{X} \end{array}$$ (7)

and similarly for *Y* and *Z*. We constructed a new SBML model based on these simplifications and generated a block configuration as shown in S4 Fig. The configuration is largely analogous to the three-stage feed-forward cascade presented earlier and repeated three times for the three gene and protein species. They differ mainly in how they account for the total variable. In the case of the repressilator, transcription factors and mRNAs regulate transcription and translation but they are not directly converted to their downstream molecules the way S is converted to T and U in the feed-forward cascade. The total variable for each biomolecule in the repressilator is not dependent on their downstream products and consequently, the degradation fluxes are not backpropagated up the circuit, as seen in the unconnected rv_up ports.

**Fig 10:**
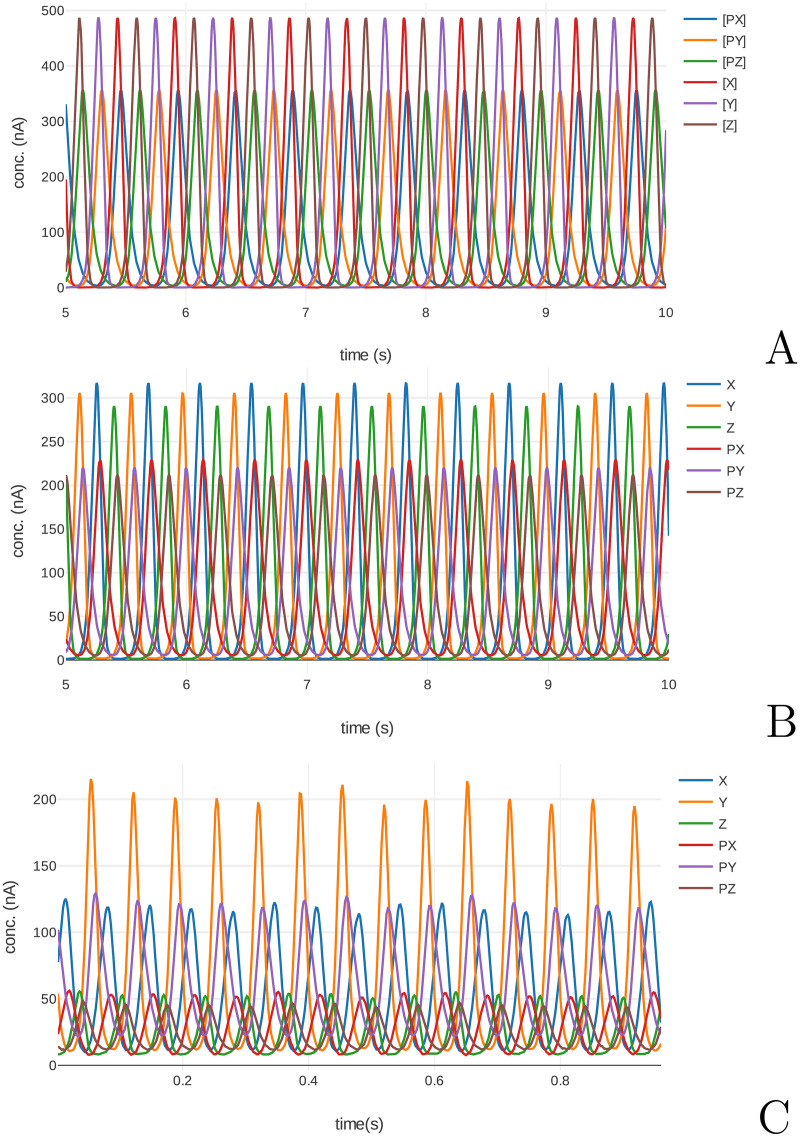
Comparison of repressilator model simulations. Also shown is an SBML simulation of the original model (A), a block simulation (B), and data collected from the cytomorphic chip (C). Due to manufacturing variations, blocks in the cytomorphic chip have different gains, hence the peak heights are different. The time axis on the chip data plot corresponds to “simulation time,” i.e. the actual duration of running the simulation, as opposed to “model time”, the time according to the dynamics of the model. To be useful, a hardware simulation should take less time to run than the timescale according to the model’s dynamics. This allows for multiple *in silico* expriments to be performed in for the amount of time a single physical experiment would take. To quantify this ratio, we performed correlation analysis on the chip data against the block simulation based on the wiring on S4 Fig (D). The cross correlation shows a peak at ≈ 6.5 seconds, indicating a six–fold difference between model and simulation time. This does not represent a speedup over a software SBML simulation, but the cytomorphic chip exhibits constant scaling up to the maximum number of blocks.

Fig 10 shows a digital SBML simulation of the repressilator model, a block simulation (emulation of the cytomorphic hardware using a digital computer), and the cytomorphic chip data for this model. The compiler is successful in configuring the wiring between blocks to produce the oscillations seen in the repressilator. The difference in oscillation amplitudes from the hardware simulation can be attributed to manufacturing variations in the chip. We hypothesized that these variations could be explained in terms of variation of the internal gains within the chip. The internal gains are the parameters ratC, KDfw, KDrv, kr, and kdeg in Table 2.

In order to compensate for these variations, we sought to determine if the gains in the digital block simulation could be adjusted to match the chip data. To perform these adjustments, we used the Nelder–Mead method with an objective function of the mean–squared–error (MSE) between the block simulation and the chip data for mRNA and protein levels. In practice, we found it sufficient to adjust only the KDfw and KDrv gains, which enabled us to reduce the MSE from 124 nA to 15.6 nA, an 87% decrease. The results of this adjustment are plotted in S7 Fig. Indeed, this strategy has been previously exploited in analog hardware for cochlear processors to correct for variation and enable deaf subjects to successfully use cochlear prosthetics on the first try [3, 49, 50].

### Higher–order compilation

While the requirement that all processes in a model be reducible to mass–action kinetics may seen restrictive, there is a very large class of models that consist only of this type of process. The field of rule–based modeling is a very active area of research (see [51] for a review). Rule–based models are composed of multi–state species. For example, a protein can have multiple phosphorylation sites, and each site can exist in either a “phosphorylated” or “unphosphorylated” state. These rules can be used to generate a network of all enumerable molecular states, or alternately simulated stochastically without enumeration using agent–like methods such those employed by the simulator NFSim [52].

Rule–based models invariably generate mass–action networks when enumerated or otherwise reduced to a simulatable form (this is not strictly a requirement, but non–mass action networks are a rare use–case for rule–based modeling and we do not consider them here). Furthermore, whereas NFSim scales linearly with the number of rules [52], the cytomorphic chip has constant scaling up to the maximum number of blocks (with the ability to connect to additional chips and thus increase the maximum network size in the future).

**Fig 11:**
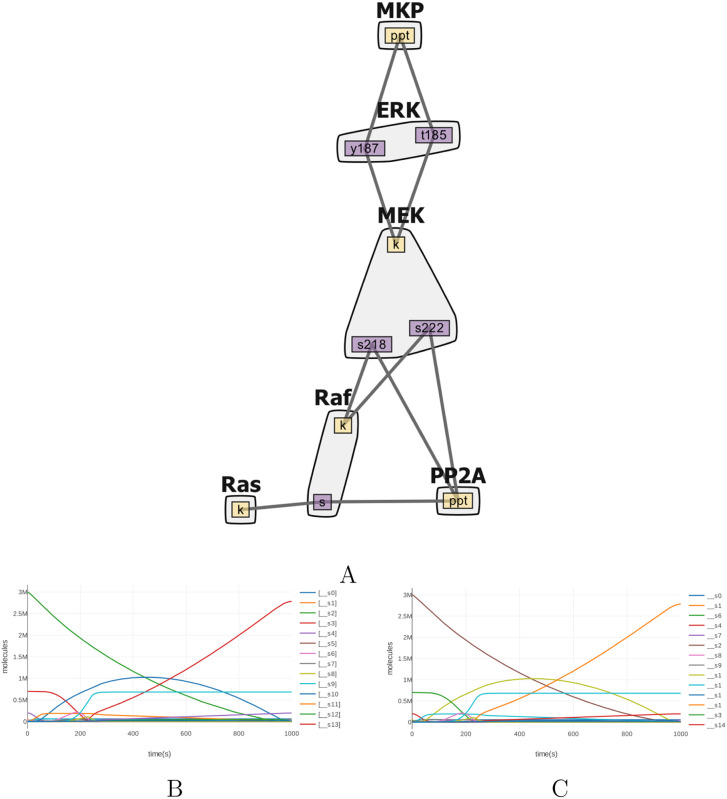
A rule–based MAP kinase model [53, 54] and its corresponding block configuration produced by the cytomorphic compiler. (A) A contact map for the kinase cascade generated using RuleBender [55]. The contact map shows the molecular species present in the model—Ras, Raf, MAPK/ERK Kinase (MEK), extracellular signal-regulated kinase (ERK), MAP kinase phosphatase (MKP), and Protein phosphatase 2 (PP2A). When expanded to an SBML mass–action network representation, this rule–based model expands into a network with 20 reactions and 21 distinct dynamical states. An SBML simulation of the flattened rule–based model (B) and a block simulation (C) are identical up to 3 significant figures (simulated using libRoadRunner, CVODES solver, absolute and relative tolerances 10^−20^ and 10^−12^ respectively).

Taken together, these factors suggest that the ideal application of cytomorphic hardware could be the simulation of rule–based mass–action networks. We therefore selected a model of a MAPK signaling cascade from the rule–based modeling platform PySB [53] and based on a previous study [54] to validate our cytomorphic compiler. This cascade consists of the MAPK ERK and its upstream activators Ras and Raf. A contact map for this model, generated using RuleBender [55], is shown in Fig 11.

The full block layout and wiring for this model is shown in S6 Fig.

Rule–based models are one approach to managing complexity. They allow the user to specify models in terms of biomolecules with multiple states (such as multiple phosphorylation sites and multiple binding sites that can either be occupied or not) and automatically enumerate all possible discrete states. Similarly, electronics designers have long used automated logic synthesis to generate chip layouts from high–level logic specifications. Just as automated placement and routing was a necessary technology for enabling rapid growth in complexity of integrated circuits (ICs), we believe technologies such as the cytomorphic compiler presented here will be necessary for rapid growth of biomimetic electronics.

### Discussion

Special–purpose hardware is necessarily designed to solve a specific subset of problems. One must sacrifice generality for the sake of improved performance and / or efficiency on this subset. Our system clearly makes this trade–off of generality for performance. In this section we discuss limitations, work–arounds, and future plans for improving the generality of the hardware and software.

The most obvious limitation of the cytomorphic hardware is that all processes are constrained to bimolecular mass–action reactions. However, a large body of rule–based models exists which are not hindered by this restriction, since rule–based models can be converted into mass–action reaction networks [51]. We have also provided an automatic method for reducing enzyme and repressor binding kinetics to mass–action networks. Nevertheless, our method requires these lumped kinetic expressions to be explicitly built–in to the compiler. Our future plans include adding support for user–defined lumped kinetic expression reductions to allow for kinetic expressions not already handled.

Other less–obvious limitations include dynamic range and forcing function support. The cytomorphic hardware exhibits less dynamic range than digital simulations because the state variables in the cytomorphic chip are physical currents. Very large values would tend to be attenuated due to limitations in the amount of current that can be used by the chip. In most cases, the overall concentration values used in the model can be rescaled without changing the model dynamics by also rescaling kinetic constants appropriately. As for forcing functions, SBML allows model quantities to be defined in terms of user–specified, potentially time–dependent expressions (called assignment rules). These rules can be used to implement time–varying input to the system. In control theory, such inputs are called *forcing functions*. This feature in SBML allows researchers to test their models against different types of time–varying input. However, the cytomorphic hardware itself cannot synthesize arbitrary waveforms, thus preventing this feature from being used. Instead, assignment rules are evaluated at the initial state of the model. Any subsequent changes are not accounted for. In theory, this could be remedied by connecting an arbitrary waveform generator to the cytomorphic hardware. We plan on exploring this possibility as the hardware matures. A similar argument applies to other SBML input–centric features (e.g. events and rate rules).

One application where a hardware implementation of an ODE solver is particularly useful is in estimating the parameters of a biological model from known experimental data. Current methods of parameter estimation iterate between model simulation using an initial set of parameters, and exploring a new set of parameters that minimizes a loss function [56]. It is widely recognized that the bottleneck in parameter estimation is the numerical integration of the system of ODE since it is repeated for every new set of parameters. Furthermore, a robust statistical approach to parameter estimation must necessarily make use of multiple solutions of an optimization problem, either as part of a Bayesian approach (e.g. [57]) or uncertainty analysis [58]. Thus, a highly parallel, high performance–per–Watt analog computer for performing many such simulations is highly sought–after.

In contrast to digital simulations, which are exact, analog simulations suffer from accuracy problems that are caused by mismatches in component resistances and capacitances due to manufacturing variations. We have shown that these variations can in principle be compensated for by adjusting the internal gains of each reaction block in the chip (S7 Fig). We hope to further develop this procedure to engineer a robust platform for parameter fitting based on analog computation.

The cytomorphic chip is analog hardware that runs in continuous-time domain. It contains noise generators that amplify thermal noise to create thermodynamically-accurate random fluctuations for biochemical reactions. These factors enable the chip to simulate multiple stochastic reactions in parallel to enjoy network–size–invariant speedups. By running the simulation on the cytomorphic chip, we can leverage the chip’s speedup to reduce the time of each solution to the parameter fitting problem [8, 9]. In more concrete terms, our current prototype consists of 20 reaction units and can simulation roughly 2 ~1–second repressilator simulations simultaneously. In contrast, the digital compute we used consumes roughly 100 Watts of power and can run approximately 30 analogous simulations per second. The cytomorphic chip can be scaled up to ~3000–fold more reaction units before reaching the same thermal envelope as the workstation, which is roughly a 200–fold improvement in performance for the same thermal envelope. Attaining this limit would require addressing significant challenges in data input / output and signal fidelity, among other considerations, but suggests that analog hardware may become an attractive platform for simulation in the future.

### Disclaimer

The content is solely the responsibility of the authors and does not necessarily represent the views of the National Institutes of Health, the Betty Moore Foundation, or the Alfred P. Sloan Foundation.

## Supporting information

S1 Fig A “fan–out” reaction occurs when multiple reactions consume the same reactant (*S* in this case). In such a case, the blocks are termed a *consumer group*. One of these blocks will compute *S*_*free*_ by subtracting its own *C*_*tot*_ value. This *S*_*free*_ value is then connected to the *A*_*tot*_ input for the second block. In addition, the second block also subtracts its own *C*_*tot*_ value from the input value of *S* (via routing from one of the inverted output ports for *C*_*tot*_). Thus, the computed value *S*_*free*_ is equal to *S*_*tot*_ minus *U*_*tot*_ (due to the feedback within the first block) and minus *V*_*tot*_ (due to the extra *C*_*tot*_ connection).
(EPS)

S2 Fig A dissociation reaction. A single block only contains a single integrator circuit, which is used to compute its main output *C*_*tot*_. When multiple outputs are present, they can, in general, possess different degradation rates and be produced and consumed by different sets of reactions. In order to account for this, the cytomorphic compiler creates two blocks for each dissociation reaction. The first block computes *T*_*tot*_ and sends its forward and reverse rates to the Cprod and Cdeg ports of the second block, which computes *U*_*tot*_. The second block uses the forward and reverse rates to compute the change in *U*_*tot*_ according to Eq 3 with *A*_*tot*_ = *B*_*tot*_ = 0. The production of *U*_*tot*_ will thus be the same as *T*_*tot*_ except each block may have a different degradation constant ratC and may be independently connected to other blocks that produce / consume either of the products.
(EPS)

S3 Fig A “fan–in” reaction occurs when multiple reactions produce the same species *U*. In such a case, the blocks are called a *producer group*. Since the cytomorphic chip uses “total” quantities for computations, these different sources for *U* need to be summed together to provide a single value for *U*_*tot*_. This is accomplished as follows: (1) designate a single block as the “main” producer of *U*. Other blocks that produce *U* will send their forward and reverse rates rate_fw and rate_rv to the main block’s Cprod and Cdeg ports resp. In turn, the main block sends its own computed value of *U*_*tot*_ as well as the total forward and reverse rates for *U* to all other blocks in the producer group via fw_tot and rv_tot. Finally, when *U* is removed from the system, the total values of its upstream nodes *S* and *T* must be adjusted accordingly. Internally, all non–main blocks in the producer group compute the total production and consumption of *U* and subtract their individual contributions to this amount (via inverting the FF_EN_sw switches in Fig 2B). This left–over amount is the amount by which the total value of the upstream nodes *S* and *T* changes as a result of external sources of *U*. This scheme, while complicated, allows total quantities for all species to be computed.
(EPS)

S4 Fig Wiring diagram of the repressilator model. To validate the cytomorphic compiler output for repressor kinetics, we constructed an SBML model containing six transcription / translation / degradation reactions (see Eq 7). The compiler transformed this SBML model into a block configuration containing nine blocks (A). Each transcription reaction is expanded to repressor binding (first column) and transcription (second column), whereas each translation / degradation reaction is represented by a single block (third column). A digital simulation of this block configuration is shown in Fig 10B.
(EPS)

S5 Fig To verify the correct time alignment ratio between the digital block simulations and chip data for the repressilator, we computed the cross correlation between the two signals as a function of the time ratio. The cross correlation shows a peak at ≈ 6.5 seconds, indicating a six–fold difference between model and simulation time. This does not represent a speedup over a software SBML simulation, but the cytomorphic chip exhibits constant scaling up to the maximum number of blocks.
(EPS)

S6 Fig The block layout of the PySB kinase cascade model compiled for the cytomorphic chip showing 30 blocks representing the 20 SBML reactions. This diagram is a Simulink model generated automatically by the compiler for validating the output.
(EPS)

S7 Fig Comparison of chip data for mRNA (left) and protein (right) and a digital SBML simulation with gain values adjusted to match the chip data. We hypothesized that the accuracy difference in the chip versus digital SBML simulation in Fig 10 was due to manufacturing variations that caused changes in the internal gains of the cytomorphic chip (ratC, KDfw, KDrv, kr, and kdeg in Table 2, as well as temporal (x–axis) and block output (y–axis) scaling). In practice, we found it sufficient to adjust only KDfw and KDrv of each block, as well as x and y–axis scaling, to attain close alignment between the chip results and the simulation. To fit the SBML simulation to the chip simulation, we used an objective function based on the MSE of the signals. The fit SBML simulation has an MSE of 15.6 nA versus the original MSE of 124 nA, an 87% decrease. The adjusted gains are shown in S2 Table.
(EPS)

S8 Fig The fitting procedure for KDfw and KDrv in S7 Fig was repeated for a different set of reaction blocks. In general, each block exhibits a different gain profile and must be separately corrected for.
(EPS)

S1 Table The KDfw and KDrv gains were adjusted for each of the nine blocks a digital simulation of the repressilator circuit to match the chip data in Fig 10. These gain adjustments refer to the simulation in S7 Fig.
(PDF)

S2 Table A second replicate of the gain adjustment method for KDfw and KDrv. S8 Fig shows the simulation results for these gains.
(PDF)
