# Supplementary material for: A compiler for biological networks on silicon chips
Source: PLoS Comput Biol. 2020 Sep 23;16(9):e1008063. doi: 10.1371/journal.pcbi.1008063 (PMC7535129; doi:10.1371/journal.pcbi.1008063)
Supplement: S1 Table — These gain adjustments refer to the simulation in S7 Fig. (PDF) [file pcbi.1008063.s009.pdf]

Table S1. Gain adjustments .

Block	KDfw ($\log_{10}$ fc)	KDrv ($\log_{10}$ fc)
1	0.33	0.51
2	0.00	0.00
3	0.04	0.03
4	-0.28	-0.31
5	0.00	-0.00
6	0.10	-0.18
7	0.31	0.61
8	0.00	0.00
9	0.28	0.17

The `KDfw` and `KDrv` gains were adjusted for each of the nine blocks a digital simulation of the repressilator circuit to match the chip data in Fig 10. These gain adjustments refer to the simulation in Fig S7.
