# Supplementary material for: A compiler for biological networks on silicon chips
Source: PLoS Comput Biol. 2020 Sep 23;16(9):e1008063. doi: 10.1371/journal.pcbi.1008063 (PMC7535129; doi:10.1371/journal.pcbi.1008063)
Supplement: S2 Table — S8 Fig shows the simulation results for these gains. (PDF) [file pcbi.1008063.s010.pdf]

Table S2. Gain adjustments .

Block	KDfw ($\log_{10}$ fc)	KDrv ($\log_{10}$ fc)
1	0.15	-0.15
2	0.00	0.00
3	-0.17	0.09
4	-0.42	-0.51
5	0.00	-0.00
6	-1.00	-1.05
7	-0.24	-0.03
8	0.00	0.00
9	-0.34	0.15

A second replicate of the gain adjustment method for `KDfw` and `KDrv`. Fig S8 shows the simulation results for these gains.
